# Adapt conservation biology teaching to address eco-anxiety in students

**DOI:** 10.1371/journal.pbio.3001774

**Published:** 2022-09-06

**Authors:** Vinícius de Avelar São Pedro, Larissa Trierveiler-Pereira, Juliano Marcon Baltazar

**Affiliations:** Centro de Ciências da Natureza, Universidade Federal de São Carlos, Campus Lagoa do Sino, Buri, São Paulo, Brazil

## Abstract

Eco-anxiety is a new and growing source of mental distress that seems to be particularly affecting the next generation of conservation biologists. Educators have an important role in helping to prevent worsening the mental health status of their students.

## Teaser

The fear of environmental doom (eco-anxiety) is a new and growing source of mental distress that could particularly affect the next generation of conservation biologists. With care and empathy, educators can help to prevent worsening the mental health of students.

The next generation of conservation biologists faces a new danger in the form of threats to their mental health. Problems like anxiety, depression, psychosis, panic disorder, and suicide affect an increasing portion of the population, and young people seem to be especially vulnerable [1]. Mental disorders in young adults are so prevalent and severe that the World Health Organization has recently created a Mental Health International College Student Initiative [2]. At this stage of life, the typical stressors of attending college (such as homesickness, new responsibilities, academic pressure, and career concerns) can cause or exacerbate mental health disorders.

The academic environment and the content and methods addressed in the teaching–learning process can positively or negatively affect students’ mental health. In this context, we can expect that recurring coverage of tragic or devastating subjects during classes could potentially increase preexisting mental health conditions. For example, in the teaching of conservation biology and related areas, the constant mention of environmental problems such as climate change, biodiversity loss, deforestation, and natural resource depletion can generate a picture of unsolvable global catastrophe that could obscure young people’s perspectives or even undermine their hopes.

**Fig 1:**
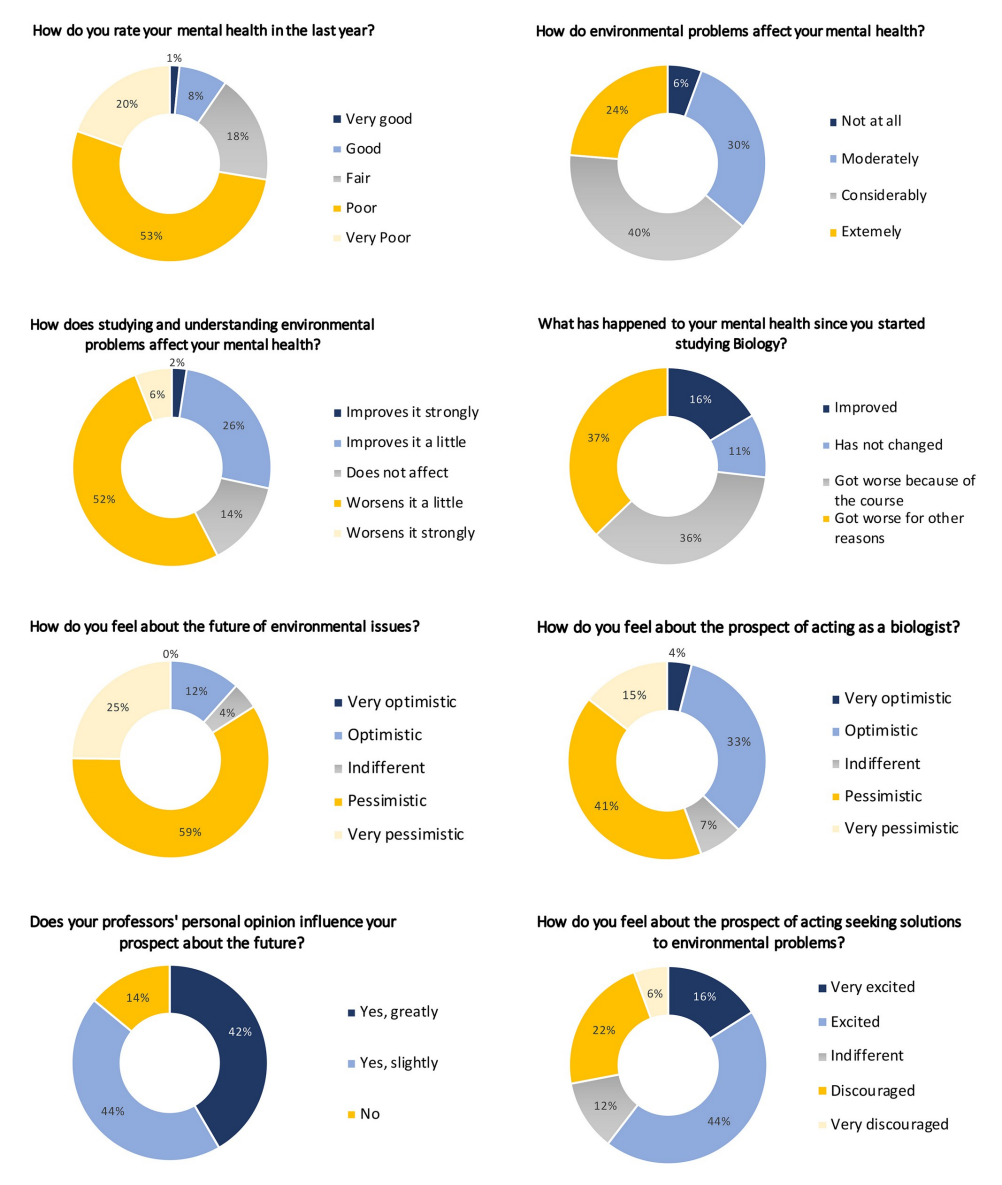
Results of a mental health questionnaire given to 250 biology undergraduate students in Brazil. Charts show the proportions of answers to questions about mental health given by 250 undergraduate students of biological sciences from 6 Brazilian public universities (UFSC, UFSCar, UFPE, UFJF, UFPI, and UENF) in March 2022.

To investigate this possibility, we undertook a mental health survey of undergraduate students studying biological sciences during March 2022. A total of 250 students from 6 Brazilian public universities (UFSC, UFSCar, UFPE, UFJF, UFPI, and UENF) replied to the questionnaire anonymously [3]. Most of the respondents considered their recent mental health to be poor and stated that environmental problems affect it to some degree (Fig 1). As expected, most of the respondents said that studying and understanding environmental problems worsened their mental health to some degree. However, despite the overall pessimism, many respondents still felt optimistic about working as biologists and the majority were excited to seek solutions to environmental problems. Importantly, the vast majority of students stated that their professors’ personal opinion influences their prospects for the future to some degree.

The students’ replies to the questionnaire suggest that a possible underlying reason for at least part of the poor mental health that the majority reported could be so-called “eco-anxiety” or “ecological grief” [4,5]. Such feelings of apprehension about anticipated threats to ecosystems and grief in relation to ecological loss are increasing rapidly among the population and demand urgent actions from public health professionals, policy makers, and educators [4]. In this respect, it is worth considering if we may be being negligent when teaching courses with a focus on conservation biology, which, not by chance, is called the “crisis discipline.” So how can we teach conservation biology without risking worsening the mental health of our students? Trying to answer this question, and based on our own classroom experiences and conversations with undergraduate students, we suggest a few strategies and actions that could be taken by environmental sciences professors and lecturers.

- Avoid being too negative. Try to use a balanced approach when dealing with critical topics, pointing to solutions among the negative findings inherent to the discipline. Try to highlight achievements from the past few years. Even if your own perspective is pessimistic, give students a chance to choose their own point of view.
- Whenever possible, try to highlight positive examples and case studies, preferably at the end of a class, to leave a positive final message.
- When there are no positive examples, try to finish the lesson by pointing out theoretical solutions to that problem. Strive to show students that those can be exciting issues for future research or engagement. Remember that many students choose a career in sciences in order to help solve environmental problems.
- Listen to the students. Create opportunities for them to express their opinions and thoughts about the topic being taught. Signs of extreme pessimism or recurring hopelessness may indicate the need for more cautious approaches. When in doubt, seek advice from mental health experts.
- Try to propose assignments and activities in which the students have to find practical solutions for environmental issues. Problem-based learning can help to improve self-confidence in future professionals [6] while making the prospect of solutions more reachable.
- Conservation biology can mix with other fields, including the arts. As a complement or alternative to assessments and teaching material, consider using literary texts, poetry, music, or paintings. Creative arts can be a valuable tool for reducing stress in students and educators [7].
- Try not to put excessive weight on students’ shoulders. Avoid passing on the message that the future of humankind rests solely upon their generation. Instead of saying things like “The burden of the environmental crisis is all yours,” try to show that “We, environmental professionals, can help change this scenario.”
- Consider if presenting certain upsetting information is really necessary. Depending on the general mood of the students at any given time, consider softening or omitting catastrophic data. In this case, do not forget to assess and mitigate possible losses of omission to educational goals.
- Choose your words carefully. Avoid using words, expressions, or figures of speech that suggest the end of the line for certain conservation issues.

As an educator, if these suggestions seem exaggerated or unreasonable to you, we recommend that you look more closely into eco-anxiety [4,5] and the current mental health epidemic among young people [8]. If you are affected by eco-anxiety yourself or feel hopeless, consider whether this is an appropriate time for you to teach this kind of discipline, particularly to undergraduate students. Talk to department colleagues and heads to find out the best solutions in such cases. If you feel that your classes may seem discouraging or depressing in some way, consider that you may also need mental health support. In this case, do not be negligent and seek help.

Young people’s mental health is a serious and urgent issue. We encourage colleagues to think about the above suggestions for teaching conservation biology and raise a discussion within their institutions and departments.

The ecological crisis might be a known source of mental health problems [5], but we also know that dispositional optimism is positively related to health and motivation [9]. Hope is an important factor in environmental engagement among young people, and educators have a vital role in activating this feeling [10]. We believe that being more emphatic and cautious in the classroom is a simple way to not only help keep our students healthy but also to ensure the necessary enthusiasm for the future protagonists of conservation biology.
